# Diffuse Alveolar Haemorrhage Complicated by Pulmonary Metastasis of Cardiac Angiosarcoma and Kasabach–Merritt Syndrome: A Case Report

**DOI:** 10.1002/rcr2.70371

**Published:** 2025-10-01

**Authors:** Takuma Ikeda, Satoru Terada, Kazuo Endo

**Affiliations:** ^1^ Department of Respiratory Medicine Hyogo Prefectural Amagasaki General Medical Center Amagasaki‐shi Hyogo Japan

**Keywords:** angiosarcoma, diffuse alveolar haemorrhage, Kasabach–Merritt syndrome, pulmonary metastasis, thrombocytopenia

## Abstract

Kasabach–Merritt syndrome (KMS) is a consumptive coagulopathy caused by platelet trapping due to the distinctive endothelial structure of vascular tumours. Cardiac angiosarcoma complicated by KMS is exceedingly rare. We report the case of cardiac angiosarcoma in which diffuse alveolar haemorrhage was caused by both pulmonary metastases and KMS. Invasive mechanical ventilation with high positive end‐expiratory pressure (PEEP) temporarily achieved transfusion‐independent haemostasis. However, a biopsy could not be performed during this window, and the patient experienced a fatal outcome. In case of cardiac angiosarcoma complicated by KMS, early tissue diagnosis is critical. Strategies to achieve transfusion‐independent haemostasis such as high PEEP may create an opportunity for prompt biopsy and facilitate timely initiation of definitive treatment.

## Graphical abstract

We report an exceptional case of cardiac angiosarcoma in which diffuse alveolar haemorrhage was caused by both pulmonary metastases and Kasabach–Merritt syndrome. Strategies to achieve transfusion‐independent haemostasis such as high PEEP may create an opportunity for prompt biopsy and facilitate timely initiation of definitive treatment.

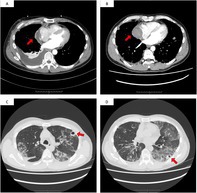

## 1 Introduction

Cardiac angiosarcoma is an exceedingly rare malignancy that most commonly originates in the right atrium, tends to metastasize early, and is unresectable in more than half of cases at the time of diagnosis. The median overall survival for advanced disease is approximately 6 months, yet systemic chemotherapy can provide meaningful benefit, highlighting the importance of prompt diagnosis and treatment [1].

Kasabach–Merritt syndrome (KMS) is characterised by the overconsumption of coagulation factors caused by proliferating vascular endothelial cells, which trap platelets and activate both the coagulation and fibrinolytic systems, leading to profound thrombocytopenia and hypofibrinogenemia [2]. Although KMS is commonly associated with Kaposi sarcoma and hepatic angiosarcoma, its association with cardiac angiosarcoma is exceptionally rare [3]. We present a rare case of cardiac angiosarcoma in which diffuse alveolar haemorrhage (DAH) was caused by both pulmonary metastases and KMS. We also explore therapeutic strategies for achieving transfusion‐independent haemostasis while facilitating early tissue diagnosis in this highly lethal clinical setting.

## 2 Case Report

**FIGURE 1:**
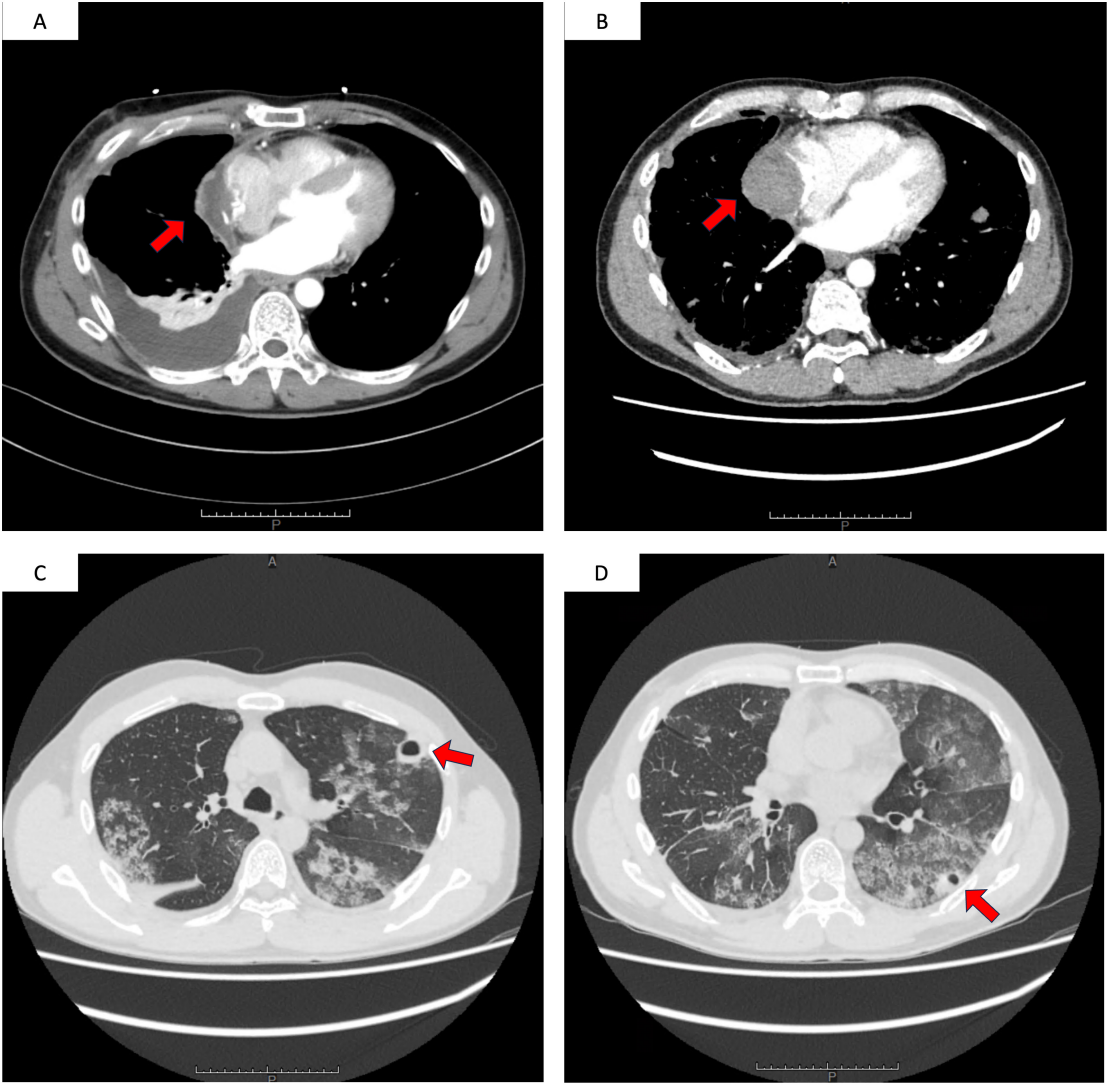
Computed tomography (CT) finding. (A) CT scan at the time of right atrial rupture 2 months earlier. High‐attenuation soft tissue lesion in the right atrium was observed. (B) CT scan at the time of presentation to our hospital. Slight enlargement of high‐attenuation soft tissue lesion in the right atrium was observed. (C and D) CT scan at the time of presentation to our hospital. Diffuse ground‐glass opacities with associated thin‐walled cystic lesions were observed in both lungs.

A 55‐year‐old man presented to our hospital with progressive dyspnoea and haemoptysis. Two months earlier, he underwent right atrial repair for idiopathic right atrial rupture, in which a fingertip‐sized defect was observed in the right atrial wall, while the surrounding atrial tissue appeared normal (Figure 1A). Cytological examination of the intraoperative specimen revealed no evidence of malignancy, and his postoperative course was uneventful.

On admission, he was alert and oriented. Blood pressure and heart rate were within normal limits, the respiratory rate was 29 breaths/min, oxygen saturation was 94% on 3 L/min oxygen via nasal cannula, and there was no fever. Physical examination revealed subconjunctival haemorrhage, gingival bleeding, and purpura on the lower extremities. Laboratory findings showed: white blood cell count 7000/μL (reference range: 3300–8600/μL); haemoglobin 8.7 g/dL (11.6–14.8 g/dL); platelet count 6 × 10^9^/L (158–348 × 10^9^/L); prothrombin activity (PT) 68% (70%–120%); PT‐INR 1.18 (0.90–1.26); activated partial thromboplastin time 30 s (24–34 s); fibrinogen 113 mg/dL (200–400 mg/dL); D‐dimer 358 μg/mL (≤ 1 μg/mL); fibrin/fibrinogen degradation products (FDP) 665 μg/mL (≤ 4 μg/mL); and C‐reactive protein 2.58 mg/dL (0.00–0.14 mg/dL). Contrast‐enhanced computed tomography (CT) revealed a high‐attenuation area in the right atrium, diffuse bilateral ground‐glass opacities with thin‐walled cysts in lung, and multiple hepatic nodules (Figure 1B–D). The right atrial lesion was initially interpreted as a postoperative hematoma or postoperative change. Based on haemoptysis, diffuse ground‐glass opacities on CT, and severe thrombocytopenia, a clinical diagnosis of DAH was made. Bronchoalveolar lavage could not be performed because of thrombocytopenia and unstable respiratory status. Empirical antibiotics were administered for suspected septic pulmonary embolism; however, blood cultures remained negative.

Despite transfusion of large volumes of platelets, with a target platelet count above 50 × 10^9^/L and fibrinogen maintained above 150 mg/dL using platelet concentrates and fresh frozen plasma, DAH progressed. Empirical steroid pulse therapy was initiated; however, DAH did not improve. On hospital day 3, the patient was intubated and managed with invasive mechanical ventilation with high positive end‐expiratory pressure (PEEP, 15 cmH_2_O), which resulted to marked improvement of DAH and obviated the need for further platelet transfusion. Extensive diagnosis workup revealed no evidence of antineutrophil cytoplasmic antibody‐associated vasculitis, fungal infection, immune thrombocytopenia, or thrombotic thrombocytopenic purpura, leaving malignancy as the only remaining differential diagnosis.

**FIGURE 2:**
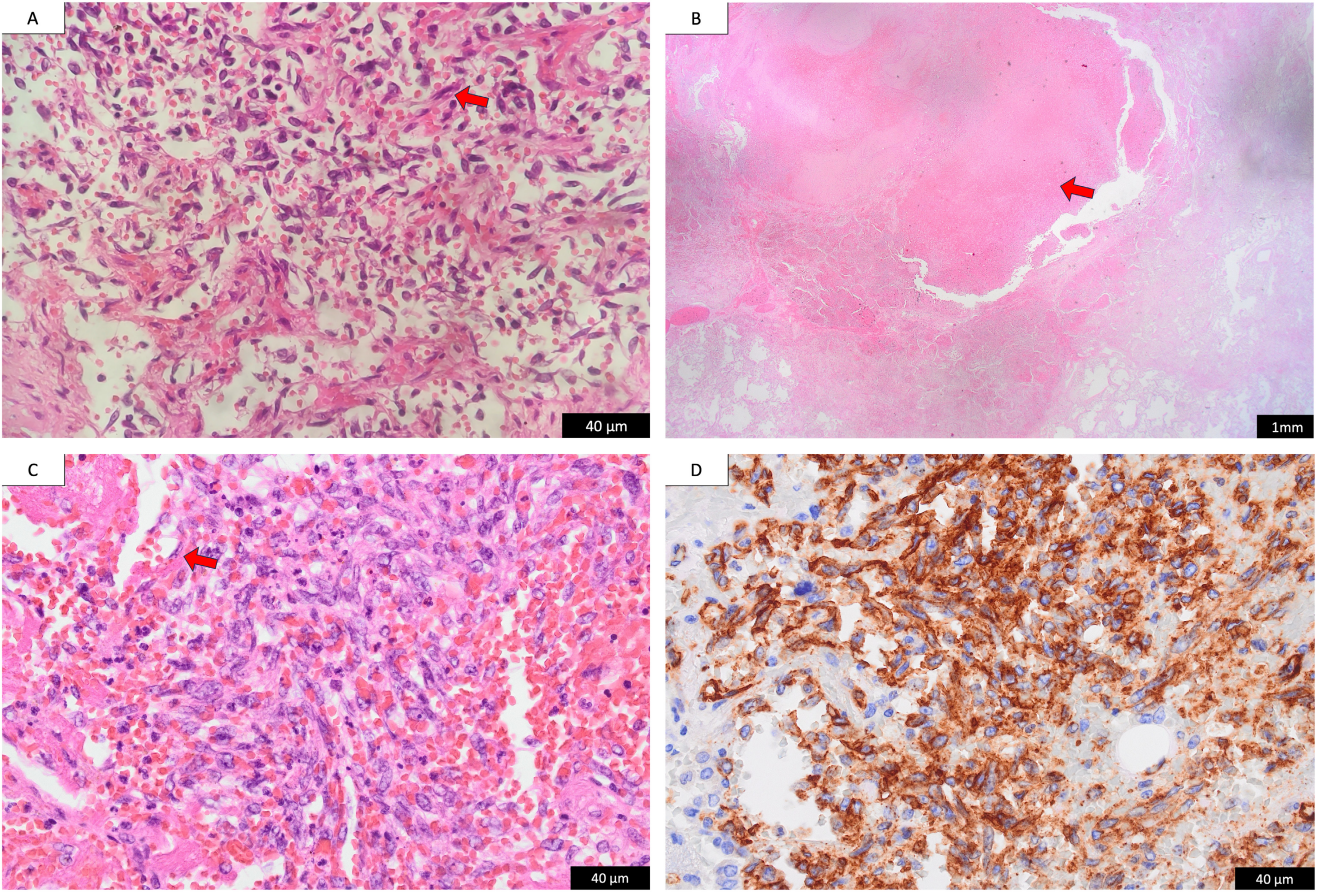
Histopathological and immunohistochemical findings. (A) Right atrium (Haematoxylin and eosin, ×400): Numerous coalescent vascular channels are proliferating, and spindle cells are present, consistent with angiosarcoma. (B) Lung (Haematoxylin and eosin, ×12.5): A nodular lesion in the centre is packed with blood, and haemorrhage extends into the adjacent alveoli. (C) Lung (Haematoxylin and eosin, ×400): Along with haemorrhage, a small number of spindle cells are observed. (D) Lung (Immunohistochemistry, ×400): CD34 is positive.

Although he was extubated on hospital day 9, DAH recurred along with worsening thrombocytopenia. His respiratory condition deteriorated, and he died on hospital day 14 (Table 1). Postmortem examination revealed widespread infiltration of spindle cells positive for CD31 and CD34 in the heart, lungs, liver, and bone marrow, leading to a diagnosis of metastatic cardiac angiosarcoma (Figure 2).

**TABLE 1:** Transition in platelet count, platelet transfusion, and respiratory management during admission.

	Day 1	Day 3 (Intubation)	Day 4	Day 7	Day 8	Day 9 (Extubation)	Day 10	Day 14
Platelet (×10^9^/L)	6	9	44	84	83	36	13	9
Transfusion volume (Unit)	20	30	10	0	0	10	20	20
Oxygen device	LFOT	LFOT → IMV	IMV	IMV	IMV	IMV → NPPV	NPPV	HFOT

Abbreviations: HFOT, high‐flow oxygen therapy; IMV, invasive mechanical ventilation; LFOT, low‐flow oxygen therapy; NPPV, noninvasive positive pressure ventilation.

## 3 Discussion

KMS is a recognised complication of angiosarcoma, but its occurrence in cardiac angiosarcoma is exceedingly rare, with only two cases reported to date, including the present case [3]. To our knowledge, this is the first reported case of cardiac angiosarcoma in which DAH was caused by both pulmonary metastases and KMS.

The mechanism of haemorrhage in pulmonary metastases of angiosarcoma is thought to involve the rupture of sinusoids within the tumour. Angiosarcomas form irregular and fragile vascular channels within the tumour, which frequently communicate with one another, creating a sinusoid‐like anastomosing network. Rupture of these immature vessels can lead to both simultaneous cyst formation and haemorrhage, radiologically manifesting as thin‐walled cysts surrounded by ground‐glass opacities on CT [4]. In the present case, similar radiological features were observed. Histological examination confirmed ruptured vascular sinusoids and hematoma formation within cysts, along with pericystic alveolar haemorrhage, suggesting tumour‐related bleeding.

In addition, the presence of KMS‐associated profound thrombocytopenia likely exacerbated the DAH. KMS is characterised by the trapping of platelets within the tumour and excessive consumption of coagulation factors, resulting in profound thrombocytopenia and hypofibrinogenemia. Laboratory findings typically include markedly elevated D‐dimer and FDP, with PT and APTT being normal or mildly prolonged [2]. These profiles were observed in this case. The coagulopathy developed in parallel with the rapid progression of angiosarcoma, consistent with the diagnosis of KMS, and the profound thrombocytopenia likely contributed to the worsening of DAH.

Angiosarcoma complicated by KMS carries an extremely poor prognosis [5]. Therefore, early intervention requires a strategy that enables haemostasis independent of platelet transfusion along with prompt diagnosis. Platelet transfusions have been reported to worsen the clinical course of KMS, highlighting the need for bleeding control at the source [2]. In this case, invasive mechanical ventilation with high PEEP achieved temporary control of DAH without the need for platelet transfusion. High PEEP may contribute to haemostasis by increasing intra‐alveolar pressure and compressing bleeding sites from within.

It is crucial to perform a biopsy during this transiently stable period to secure a definitive diagnosis and initiate treatment. In this case, intraoperative cytology failed to reveal malignant cells, likely because cytological examination has limited sensitivity for angiosarcoma. In patients with a history of idiopathic cardiac rupture and multiple thin‐walled pulmonary cysts, cardiac angiosarcoma should be suspected, and adequate tissue biopsy should be performed without delay. In the present case, the opportunity for biopsy was missed while diagnostic efforts were focused on pursuing alternative diagnoses. We propose that invasive mechanical ventilation with high PEEP may serve as an effective approach to achieve transfusion‐independent haemostasis, facilitate timely biopsy, and enable a smooth transition to definitive therapy in life‐threatening conditions.

## Author Contributions

Takuma Ikeda contributed to patient management, data collection, and drafted the initial manuscript. Satoru Terada and Kazuo Endo supervised clinical care, contributed to the conception of the work, and critically revised the manuscript. All authors reviewed the final version of this manuscript and approved it to be published.

## Consent

The authors declare that written informed consent was obtained for the publication of this manuscript and accompanying images using the consent form provided by the Journal.

## Conflicts of Interest

The authors declare no conflicts of interest.

## Data Availability

The data that support the findings of this study are available from the corresponding author upon reasonable request.
